# Exploring Potential Determinants of Mosquito Net Ownership and Use in Somalia: A Cross-Sectional Study

**DOI:** 10.3390/diseases10040109

**Published:** 2022-11-20

**Authors:** Mitsuaki Hirai, Usman Umar, Patricia Darikwa, Ali Abdirahman Osman, Abdirahman Mohamed, Ahmed Mohamed Jama, Carolyn Masheti, Millhia Kader

**Affiliations:** 1 UNICEF, Mogadishu, Somalia; 2 Federal Ministry of Health, Mogadishu, Somalia

**Keywords:** malaria, public health, mosquito, prevention

## Abstract

In Somalia, malaria remains a major public health threat. Understanding what influences the ownership and use of mosquito nets is of vital importance to accelerate malaria prevention efforts in the country. To explore the potential determinants of mosquito net ownership and use, this study conducted a secondary data analysis of the Somalia Micronutrient Survey 2019. Survey participants were identified through the multi-stage stratified cluster sampling, and logistic regression was performed for bivariate and multivariate analysis. The results suggested that household head’s age, educational attainment, household size, employment status of household members, socioeconomic status, geographic regions and type of residence are significantly associated with mosquito net ownership (*p* < 0.05). The analysis also highlighted household head’s age as an influential factor to mosquito net use. By further exploring and understanding the psychosocial determinants of mosquito net ownership and use, malaria prevention interventions can be made more effective in Somalia.

## 1. Introduction

Malaria is an infectious disease caused by protozoan parasites which belong to the genus *Plasmodium* and can be transmitted by female *Anopheles* mosquitos [1,2]. Despite a major reduction in malaria morbidity and mortality in the past decades [3,4,5], malaria remains the sixth leading cause of under-five mortality [6]. The COVID-19 pandemic also disrupted health services for malaria prevention and treatment, and it led to the increase in malaria cases from 227 million in 2019 to 241 million in 2020 [7]. Malaria deaths also increased from 558,000 to 627,000 during the same time period [7].

Use of insecticide-treated nets (ITNs) including long-lasting insecticidal nets (LLINs) is a cost-effective intervention to prevent malaria [8,9]. ITNs have been distributed through mass campaigns and continuous distributions at fixed locations such as health facilities and schools [10]. Since 2004, approximately 2 billion ITNs have been provided to Sub-Saharan Africa (SSA), and 65% of households had access to at least one ITN by 2020 [7].

Previous studies examined the potential determinants of long-lasting insecticidal net (LLIN) ownership and use in SSA countries such as Zimbabwe, Rwanda, and Ethiopia [11,12,13]. The findings from these studies revealed that LLIN ownership was significantly associated with household head or respondent’s biological sex, age, education levels, awareness of LLIN as a preventive measure, household assets and characteristics (e.g., type of roof, type of cooking fuel, availability of electricity), household wealth levels, geographic regions, and type of residence. LLIN utilization was associated with household head’s age and sex, household assets and characteristics (e.g., number of sleeping rooms, mosquito net hanged), pregnancy, household wealth levels, awareness of LLIN, and type of residence. Accordingly, demographic, socio-economic, and geographic or environmental factors have been identified as key determinants of LLIN ownership and use.

The United Nations Children’s Fund (UNICEF) is supporting the mass distribution of LLINs in Somalia in 2022. Understanding what influences the ownership and use of LLINs is of vital importance to accelerate malaria prevention efforts in Somalia. Few studies, however, have been conducted to characterize and explore malaria prevention practices in the country. To fill the knowledge gap and inform future malaria interventions, this study explored what factors are associated with mosquito net ownership and use in Somalia.

## 2. Methods

### 2.1. Study Context

Somalia is a malaria-endemic country in Sub-Saharan Africa with over 15.8 million people, and 51% of the population is estimated to live in a high transmission area (e.g., more than one case per population of 1000) [7,14]. WHO estimated that in 2020, Somalia had 829,649 malaria cases (491,000–1,247,000) and 2123 deaths (57–4730) [7]. From 2005 to 2019, malaria incidence declined from 136.6 to 49.2 per population of1000 [15]. Over 1.5 million LLINs were sold or delivered in 2020 in the country [7].

### 2.2. Data Source

This study analyzed secondary data from the Micronutrient Survey 2019, a cross-sectional household survey that mainly aimed to explore the nutritional status of women and children and their living conditions in Somalia. As with the major household surveys (e.g., Demographic and Health Survey, Multiple Cluster Indicator Survey), the Micronutrient Survey used the multi-stage stratified cluster sampling. More specifically, this survey included six strata (1. Somaliland, 2. Puntland, 3. Hirshabelle and Galmudug, 4. Jubaland and South-West, 5. Banadir, and 6. internally displaced populations [IDP] camps in the aforementioned strata). For the first five strata, 25 clusters or primary sampling units were randomly selected with probability proportional to size. For the IDP camp selection, this survey referred to UNHCR’s IDP database to determine the sampling frame, which included all IDP camps and household information. Based on the available household lists, 16 households were randomly selected in each cluster. Additional information on the sampling procedure and sample size calculation is described elsewhere [16,17]. The survey teams visited a total of 2,400 households and collected data from 2172 households with a response rate of 90.5%.

### 2.3. Variables

**Figure 1:**
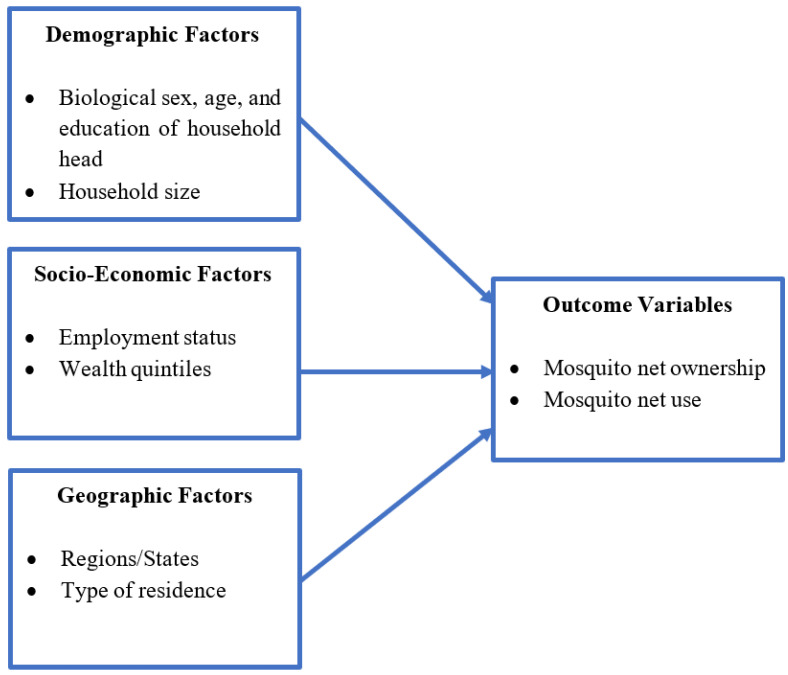
A conceptual model on the potential relationship among key variables.

The main dependent variables are the household ownership of at least one mosquito net (1. No, 2. Yes) and the household utilization of all mosquito nets owned in the previous night (1. No, 2. Yes). The independent variables consist of demographic, socioeconomic, and geographic factors (Figure 1). Demographic variables include household head’s biological sex (1. Male, 2. Female), age group (1. 25 years old or younger, 2. 26 to 35 years old, 3. 36 to 45 years old, 4. 46 years old or older), educational level (1. None or pre-school, 2. Koranic, 3. Primary school or higher), household size (1. 1 to 3 people, 2. 4 to 7 people, 3. 8 or more). Socioeconomic variables are employment status of household members (1. No household member with an employment, 2. Any household member with an employment) and wealth quintile (1. Poorest, 2. Poorer, 3. Middle, 4. Richer, 5. Richest). The wealth quintile was estimated through the principal component analysis of household characteristics such as type of water source, sanitation facility, livestock ownership, materials used for floors and walls). Geographic variables consist of states (1. Banadir, 2. Somaliland, 3. Puntland, 4. Hirshabelle, 5. Galmudug, 6. Southwest, 7. Jubaland) and type of residence (1. Rural, 2. Urban, 3. IDP camps).

### 2.4. Data Analysis

Univariate analysis was conducted to characterize the frequency of each study variable. For bivariate analysis, simple logistic regression was conducted to assess the association between independent and dependent variables. The F statistic and *p*-value of 0.05 were used to assess statistical associations between study variables. For multivariate analysis, multiple logistic regression was conducted to estimate the way each independent variable was associated with a dependent variable by holding other variables constant. The results of bivariate and multivariate analysis were presented in odd ratios. All analyses were performed with STATA 14, adjusting for the complex survey design.

### 2.5. Ethical Approval

This study used secondary data without any personally identifiable information. Thus, it did not require ethical approval. However, the original Micronutrient Survey 2019 obtained ethical approval from the Federal Ministry of Health and Ministries of Health in Puntland and Somaliland. The household-level data were obtained from the household heads (or spouse/other adult members in case of their absence) who provided written informed consent to participate in the survey. For those household heads who could not read and write at the time of data collection, the informed consent form was read out loud, and a fingerprint was collected as evidence of consent.

## 3. Results

Table 1 presents the household characteristics of survey participants. At least 87% of households were female headed, and the majority of household heads were 35 years old or younger. Over 75% of household heads received no formal education or preschool education, and less than 14% of them completed primary education or higher. Approximately half of households had up to three household members, and the other half had four or more household members. About 33% of households had at least one person who was employed with income. The wealth quintiles were distributed at 20%. The representation of states ranged from Hirshabelle at 4.5% to Somaliland at 26.4%. About one third of households were located in rural areas, while 49% of households were from urban areas. Over 15% of households were from IDP camps. The majority of households owned at least one mosquito net, and of those households with at least one mosquito net, 92.3% of households reported that they used all mosquito nets in the previous night.

Table 2 presents the summary of bivariate analysis between study variables. The biological sex of household heads was not significantly associated with the ownership of mosquito net (*p* = 0.222) or the full utilization of mosquito nets in the previous night (*p* = 0.637). The age and educational attainment of household head, household size, employment status of household members, wealth quintiles, states, and type of residence were significantly associated with the ownership of mosquito net (*p* < 0.05). For mosquito net use, the educational attainment of household head, employment status, and states had a significant association (*p* < 0.05).

Table 3 presents the results of multiple logistic regression on the ownership of mosquito net. The odds of having at least one mosquito net among households whose heads were 26–35 years old and 36–45 years old at the time of survey is 2.25 times and 2.09 times that of households whose heads were 25 years old or younger (*p* < 0.001). The household heads who received Koranic education are associated with 33.6% lower odds of having any mosquito net than those of household heads without any formal education or with pre-school education only (*p* < 0.025). Large households are associated with a 38.9% lower odds of mosquito net ownership than those of small households (*p* = 0.035).

Having at least one household member with an employment or income is associated with 48.0% higher odds of mosquito net ownership than those of households without any employed individual (*p* = 0.020). Compared to the households in the lowest wealth quintile, households with higher quintiles are associated with significantly higher odds of mosquito net ownership. The odds of having at least one mosquito net for the highest wealth quintile is 12.82 times those of the lowest wealth quintile (<0.001).

Compared to the households in Banadir, households in other states have a significantly higher odds of mosquito net ownership. The odds of mosquito net ownership in Hirshabelle, for instance, are approximately 3.93 times those of Banadir (*p* < 0.001). Urban households are associated with 92.5% higher odds than those of rural households (*p* = 0.013).

Table 4 summarizes the results of multiple logistic regression on the use of household-owned mosquito nets in the previous night. Compared to the households whose heads were 25 years old or younger, households with older household heads were associated with lower odds of using all mosquito nets in the previous night. More specifically, the households whose heads were 26 to 35 years old, 36 to 45 years old, and 46 years old or older had 55.5% (*p* = 0.027), 55.0% (*p* = 0.042), and 63.4% (*p* = 0.006) lower odds of mosquito net use, respectively. The odds of mosquito net use in Galmudug and Southwest were 69.7% and 78.7% lower than those of Banadir, respectively (*p* = 0.002).

## 4. Discussion

This is one of the first quantitative studies with a nationally representative sample that explored the potential determinants of mosquito net ownership and use in Somalia. The results suggested that household head’s age, educational attainment, household size, employment status of household members, socioeconomic status, geographic regions and type of residence are significantly associated with mosquito net ownership. The analysis also highlighted household head’s age as an influential factor in mosquito net use.

In this study, over 42% of households did not confirm mosquito net ownership, suggesting that they would not be able to reduce the risk of malaria and other vector-borne diseases such as dengue and yellow fever [18]. Although 57% of households reported the ownership of at least one mosquito net, promoting mosquito net ownership and use remains vital to protecting the population in Somalia. Given that 75% of household heads reported to have no formal education, public health interventions may be planned for people with varying literacy levels.

As with previous research [11,12,13], this study revealed that household ownership and use could be influenced by different factors. The age of household heads was positively associated with mosquito net ownership. However, it was negatively associated with mosquito net use. This finding suggests that families with older household heads may have more opportunities to obtain mosquito nets, but they may not use them as opposed to other families with younger household heads. The household wealth level and employment status were also key determinants of mosquito net ownership, but they did not influence mosquito net use. Addressing individual perceptions, behavioral intention, and social norms would be vital to further understanding of these findings and translating mosquito net ownership into use [19,20,21].

The findings on geographic regions and states also provided insights into the regions where malaria prevention efforts can be accelerated. The multivariate analysis revealed that households in Somaliland, Puntland, Hirshabelle, Galmudug, Southwest and Jubaland were associated with higher odds of owning mosquito nets than those in Banadir. As one of the most populated regions in Somalia, Banadir may be considered for additional interventions to enhance mosquito net ownership at the household level. Galmudug and Southwest states may also implement behavioral change communication interventions to enhance mosquito net use. Despite the current challenges with estimating malaria burden in Somalia, some regions—Bay, Lower Shabelle, and Gedo—have been associated with high malaria incidence [22]. The findings of this study and sub-national data on malaria incidence may be reviewed together to better compile the locations where malaria interventions can be implemented as priority areas.

This study noted a number of limitations and opportunities for future studies. First, limited malaria-related data were available in this study because the purpose of Micronutrient Survey 2019 was to collect in-depth information on maternal and child nutrition. Future studies and surveys may collect additional data on intrapersonal and psychosocial factors (e.g., motivation, perceived importance) to understand what additional factors influence mosquito net ownership and use in Somalia. Second, the findings of this study are not generalizable at the district level. To better inform malaria interventions with geographic priorities, additional data collection and quantitative analysis at the district level may be needed. Third, self-reported data of mosquito net ownership and use may be subject to social desirability bias [23]. Due to this potential challenge, the true percentage of mosquito net ownership and use may be lower. By collecting self-reported data with direct observations, this risk may be mitigated in the future. Lastly, the Micronutrient Survey 2019 did not specifically assess the ownership and use of ITNs or LLINs but was only able to characterize if households owned and used any type of mosquito nets as a proxy measure. Future studies may directly assess the ownership and use of ITNs and LLINs in Somalia to explore their determinants.

## 5. Conclusions

Despite the limitations, this study revealed some of the key household characteristics as the potential determinants of mosquito net ownership and use in Somalia. To enhance mosquito net ownership in Somalia, public health programs may focus on providing mosquito nets to households with a low socioeconomic status, without any employed household members, with a large household size, with a young household head, and in rural areas. Use of mosquito nets may be facilitated through behavior change communication with older household heads. By further exploring and understanding the psychosocial determinants of mosquito net ownership and use, malaria prevention interventions can be more effective in Somalia.

## Figures and Tables

**Table 1:** Sample characteristics.

Variables	n	Weighted %
Sex of Household Head		
Male	262	12.87
Female	1910	87.13
Age of Household Head		
25 or younger	491	22.01
26–35	627	28.37
36–45	399	18.83
46 or higher	655	30.79
Education of Household Head		
None or preschool	1569	75.49
Koranic	257	10.53
Primary or higher	334	13.98
Household Size		
Small (1–3 people)	1034	49.96
Medium (4–7)	883	38.25
Large (8 or more)	255	11.78
Any Employed Household Member		
Yes	792	32.99
No	1373	67.01
Wealth Quintile		
Poorest	286	20.25
Poorer	336	19.75
Middle	485	20.01
Richer	573	20.03
Richest	492	19.95
Regions/States		
Banadir	458	14.88
Somaliland	455	26.44
Puntland	343	9.37
Hirshabelle	163	4.51
Galmudug	249	7.47
Southwest	217	15.97
Jubaland	287	21.36
Type of Residence		
Rural	700	34.82
Urban	1129	49.48
IDP	343	15.70
Own At Least One Mosquito Net		
Yes	1290	57.26
No	818	42.74
All Mosquito Nets Used		
Yes	1186	92.3
No	104	7.7

**Table 2:** Bivariate associations between study variables. * n = 2108 for ownership and n = 1290 for all variables except for education of household head (n = 2096 for ownership, n = 1283 for utilization) and employment (n = 2101 for ownership, n = 1289 for utilization). Notes: Bolded = *p*-value < 0.05.

	Ownership *		All Nets Used *	
Study Variables	F	*p*-value	F	*p*-value
Sex of Household Head	1.51	0.222	0.22	0.637
Age of Household Head	10.58	**<0.001**	1.79	0.153
Education of Household Head	10.74	**<0.001**	5.60	**0.005**
Household Size	3.14	**0.046**	1.15	0.320
Any Household Member with Employment	28.96	**<0.001**	6.67	**0.011**
Wealth Quintiles	18.85	**<0.001**	1.26	0.290
States	5.13	**<0.001**	3.02	**0.009**
Type of Residence	11.59	**<0.001**	0.54	0.582

**Table 3:** Results of multiple logistic regression on mosquito net ownership. n = 2091. Notes: Bolded = *p*-value < 0.05.

	Odds Ratio	Std. Err.	t	*p*-Value	95% CIs	
Sex of Household Head (Ref: Male)						
Female	0.924	0.163	−0.45	0.657	0.652	1.310
Age of Household Head (Ref: 25 or younger)						
26–35	2.247	0.368	4.94	**<0.001**	1.625	3.105
36–45	2.087	0.432	3.55	**0.001**	1.386	3.143
46 or older	1.312	0.213	1.67	0.097	0.952	1.810
Education of Household Head (Ref: None or preschool)						
Koranic	0.664	0.119	−2.28	**0.024**	0.466	0.947
Primary or Higher	0.923	0.143	−0.52	0.603	0.680	1.252
Household Size (Ref: Small)						
Medium	0.835	0.122	−1.23	0.219	0.625	1.115
Large	0.611	0.141	−2.13	**0.035**	0.387	0.965
Any household member with employment (Ref: No)						
Yes	1.480	0.246	2.36	**0.020**	1.065	2.057
Wealth quintiles (Ref: Poorest)						
Poorer	2.264	0.582	3.18	**0.002**	1.362	3.763
Middle	4.776	1.503	4.97	**<0.001**	2.564	8.897
Richer	9.919	3.583	6.35	**<0.001**	4.857	20.256
Richest	12.822	4.721	6.93	**<0.001**	6.193	26.547
Regions/States (Ref: Banadir)						
Somaliland	2.388	0.643	3.23	**0.002**	1.402	4.066
Puntland	2.132	0.536	3.01	**0.003**	1.297	3.506
Hirshabelle	3.930	1.213	4.44	**<0.001**	2.135	7.233
Galmudug	11.376	5.617	4.92	**<0.001**	4.287	30.190
Southwest	3.580	1.119	4.08	**<0.001**	1.930	6.641
Jubaland	4.303	1.715	3.66	**<0.001**	1.957	9.460
Type of Residence (Ref: Rural)						
Urban	1.925	0.500	2.52	**0.013**	1.152	3.217
IDP	1.170	0.366	0.50	0.616	0.631	2.170
Constant	0.061	0.027	−6.24	**<0.001**	0.025	0.148
F-statistic (degrees of freedom)	6.49 (21, 123)					

**Table 4:** Results of multiple logistic regression on mosquito net use in the previous night among households with at least one mosquito net. n = 1283. Notes: Bolded = *p*-value < 0.05.

	Odds Ratio	Std. Err.	t	*p*-Value	95% CIs	
Sex of Household Head (Ref: Male)						
Female	1.089	0.454	0.21	0.837	0.478	2.482
Age of Household Head (Ref: 25 or younger)						
26–35	0.445	0.161	−2.23	**0.027**	0.217	0.912
36–45	0.450	0.175	−2.06	**0.042**	0.209	0.970
46 or older	0.366	0.132	−2.79	**0.006**	0.180	0.747
Education of Household Head (Ref: None or preschool)						
Koranic	0.650	0.271	−1.03	0.303	0.286	1.481
Primary or Higher	1.075	0.465	0.17	0.867	0.457	2.528
Household Size (Ref: Small)						
Medium	1.003	0.275	0.01	0.992	0.583	1.724
Large	0.672	0.282	−0.95	0.345	0.293	1.541
Any household member with employment (Ref: No)						
Yes	0.677	0.242	−1.09	0.277	0.334	1.372
Wealth quintiles (Ref: Poorest)						
Poorer	2.706	1.590	1.69	0.092	0.847	8.647
Middle	0.746	0.437	−0.50	0.618	0.234	2.377
Richer	0.648	0.411	−0.68	0.495	0.185	2.270
Richest	0.309	0.201	−1.81	0.073	0.086	1.116
Regions/States (Ref: Banadir)						
Somaliland	1.849	0.782	1.45	0.148	0.801	4.266
Puntland	1.307	0.724	0.48	0.630	0.437	3.907
Hirshabelle	1.043	0.605	0.07	0.942	0.331	3.287
Galmudug	0.303	0.113	−3.20	**0.002**	0.145	0.634
Southwest	0.213	0.105	−3.14	**0.002**	0.081	0.565
Jubaland	0.728	0.425	−0.54	0.588	0.230	2.310
Type of Residence (Ref: Rural)						
Urban	0.991	0.347	−0.03	0.980	0.496	1.982
IDP	0.646	0.292	−0.97	0.335	0.265	1.578
Constant	74.558	57.093	5.63	<0.001	16.399	338.965
F-statistic (degrees of freedom)	4.47 (21, 116)					

## Data Availability

The data presented in this study is available upon request from the corresponding author.
